# Thyroid hormone receptor subtype-specific function in controlling organ-specific developmental timing and rate during *Xenopus* development

**DOI:** 10.3389/fendo.2025.1614439

**Published:** 2025-06-09

**Authors:** Yuta Tanizaki, Yun-Bo Shi

**Affiliations:** ^1^ Department of Life Sciences, Graduate School of Arts and Sciences, The University of Tokyo, Tokyo, Japan; ^2^ Section on Molecular Morphogenesis, Eunice Kennedy Shriver National Institute of Child Health and Human Development (NICHD), National Institutes of Health (NIH), Maryland, MD, United States

**Keywords:** Xenopus laevis, Xenopus tropicalis, developmental timing, metamorphosis, thyroid hormone receptor, chromatin remodeling, transcriptional regulation

## Abstract

Thyroid hormone (T3) is essential for vertebrate development as animals fail to develop into adults in the absence of T3. T3 is particularly critical for postembryonic development. This is a period around birth in mammals when most organs mature as plasma T3 level peaks. Unlike embryogenesis, postembryonic development has not been well-studied in mammals due to the difficulty to manipulate mammalian embryos and neonates. In contrast, anuran metamorphosis involves drastic transformations of essentially every organ/tissue of a tadpole and can be easily manipulated externally without maternal influence. In addition, most changes during metamorphosis resemble organ-maturation during postembryonic mammalian development. Thus, metamorphosis offers a unique and highly advantageous opportunity for studying postembryonic vertebrate development. Studies on the metamorphosis of *Xenopus laevis* and *Xenopus tropicalis*, two highly related species have offered significant insights on the function of thyroid hormone receptors in development. Here we will review some of these studies, with particular emphasis on recent genetic and genome-wide molecular analyses in the diploid species *Xenopus tropicalis*, that support a dual function model of TR, involving distinct, organ-specific roles of TRα and TRβ, the only known TR genes in all vertebrates.

## Introduction

**Figure 1:**
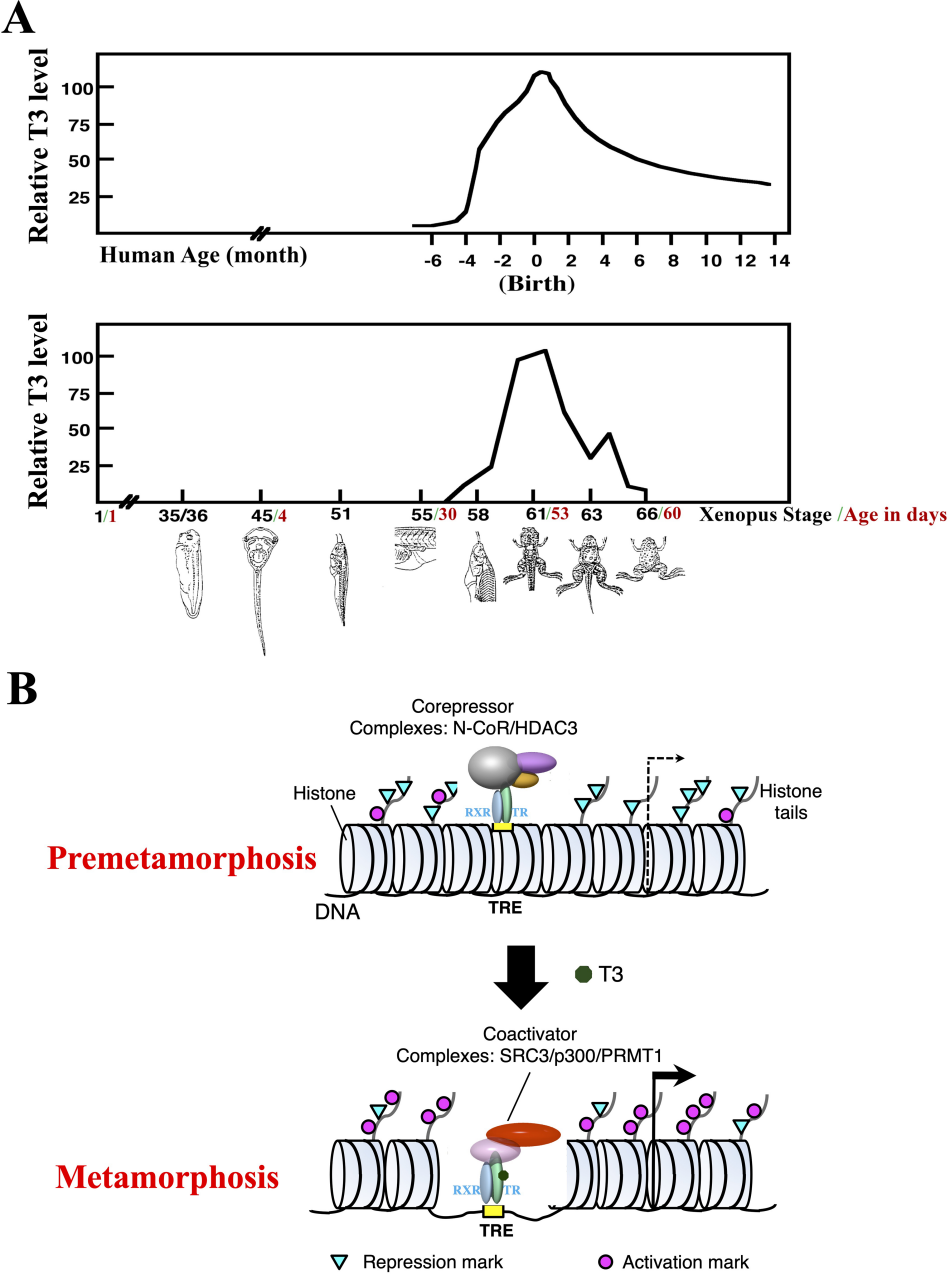
T3 is critical for postembryonic development by regulating transcription through TR. **(A)** Plasma T3 levels during human and *Xenopus* development (with peak level set to 100 for each species). Frog metamorphosis, which takes place between stage 54 to stage 66, roughly the second month of *Xenopus* development, transforms essentially every single tissue/organ of a tadpole as the animal changes into a frog. Metamorphosis shares many similarities with postembryonic development in human, a period of about 4 months before to several months after birth when most organs/tissues mature into adult forms. Of note, plasma T3 level peaks during both metamorphosis in frog and postembryonic development in human (1). **(B)** A dual function model for TR during *Xenopus* metamorphosis. During premetamorphosis (before stage 54), there is little or no T3 present and thus most TRs are in the unliganded state and TR/RXR heterodimers bind to TREs in chromatin to recruit HDAC-containing corepressors complexes to target genes. This leads to reduced levels of activation histone marks and increased levels of repression histone marks to facilitate repression of the target genes. During metamorphosis, T3 binds to TRs, which in turn releases corepressor complexes and recruit coactivator complexes to cause chromatin remodeling, including the loss of 2–3 nucleosomes around the TRE and changes in histone modifications, e.g., increased activation histone marks and decreased repression histone marks, to facilitate transcriptional activation of target genes.

Thyroid hormone (T3) is essential for vertebrate development. In the absence of T3, animals fail to develop into adults. In mammals, T3 is critical for postembryonic development, a period around birth when plasma T3 level peaks (**Figure 1A**) and many organs mature into their adult forms (1). T3 deficiency during this period lead to developmental defects that can cause life-long diseases or abnormalities. On the other hand, it is difficult to study postembryonic development in mammals due to in part the maternal dependence of the uterus-enclosed embryos and even neonates. Interestingly, most changes during mammalian postembryonic development resemble those taking place during anuran metamorphosis when a tadpole is transformed into a frog as plasma T3 level also peaks (**Figure 1A**) (1–5). Importantly, anuran metamorphosis is independent of maternal influence and can be easily manipulated with exogenous T3 or T3 synthesis inhibitors added to the tadpole rearing water. Furthermore, T3 can also induce the same metamorphic changes when added to cultures of many premetamorphic tadpole organs, suggesting that individual tissues/organs are genetically programmed to undergo specific changes in response to T3. These make metamorphosis an easy and valuable model to study not only organ transformations during postembryonic development but also likely evolutionarily conserved function of T3 and corresponding underlying mechanisms during vertebrate development *in vivo*.

T3 functions by regulating target gene transcription through its nuclear receptors or T3 receptors (TRs) (6–18). TRs are sequence-specific DNA binding transcription factors that can heterodimerize with 9-cis retinoic acid receptors (RXRs). For T3-inducible genes, TR/RXR heterodimers can bind to T3-response elements (TREs) with a consensus sequence of two direct repeats of AGGTCA separated by 4 base pairs (DR4 TREs) even in chromatin (**Figure 1B**). In the absence of T3, this binding leads to the recruitment of histone deacetylase (HDAC)-containing corepressor complexes, such as N-CoR (nuclear corepressor) complexes, that help to establish a repressive chromatin environment to repress the expression of the genes below the basal level in the absence of TR. When T3 is present, the corepressor complexes are released, and coactivators are recruited by liganded TR to the target site. The coactivator complexes include those containing histone methyltransferases, such as PRMT1 (protein arginine methyltransferase 1), and histone acetyltransferases such as SRC3 (steroid receptor coactivator 3) and p300, chromatin remodeling complexes, and/or the Mediator complex that bridges transcription factors bound to DNA at enhancers to the transcriptional machinery at promoters. Thus, TR may affect animal development by either repressing gene expression through corepressors or activating transcription though coactivators depending upon the availability of T3.

## Dual functions of TR during *Xenopus* development

The contrasting functions of unliganded and liganded TR together with their developmental expression profile and plasma T3 levels led to the proposal of a dual function model for TR during *Xenopus* development (6, 19) (**Figure 1B**). That is, for T3-inducible genes, they are expressed at a basal level during embryogenesis (stage 1 to stage 45, the onset of tadpole feeding, **Figure 1A**) when there is little TR or T3 present. After stage 45, the increased expression of TR, mainly TRα, and RXR, mainly RXRα, leads to the formation of TR/RXR heterodimers that can bind to the target genes to repress their expression as there is little T3 present prior to stage 54, the onset of metamorphosis (**Figure 1B**). This repression helps to control metamorphic timing and thus ensure a proper period of tadpole growth and development before initiating metamorphosis around stage 54 when endogenous T3 level rises. During metamorphosis, T3 binds to TR, causing the release of corepressors and recruitment of coactivators (**Figure 1B**). This in turn results in changes in histone modification and chromatin remodeling. This then activates target gene transcription to promote metamorphosis.

## Molecular and transgenic evidence supporting the model

Over the years, various molecular and genetic studies by different laboratories have provided strong evidence to support this model, initially carried out in *Xenopus laevis*, the pseudo-tetraploid species well established for various molecular, cell biological, and developmental studies. First, chromatin immunoprecipitation (ChIP)-assays showed that TR and RXR were indeed bound to TR-target genes in both premetamorphic and metamorphosing-tadpoles but little or no binding were detected during embryogenesis (20). Importantly, ChIP-assay also showed that corepressors were recruited by TR to target genes in premetamorphosis tadpoles but released during metamorphosis or when premetamorphic tadpoles were treated with T3 (21–23). The opposite was true with coactivators (24–28). Accompanying these changes in cofactor recruitments were changes in histone modification and chromatin remodeling (20, 29–31). The repression histone marks, which are associated with gene repression, and activation histone marks, which are associated with gene activation, were reduced and increased, respectively, at target genes during metamorphosis or when premetamorphic tadpoles were treated with T3. Furthermore, ChIP-assays also showed that total histone association with TRE regions were reduced to the amount equivalent to the loss of 2-3 nucleosome at each TRE site. Interestingly, such a loss is identical to the finding from earlier studies in the reconstituted *Xenopus* oocyte transcription system to study the effects on chromatin by T3 and TR on the injected plasmid reporter DNA assembled into chromatin *in vivo*. Thus, the dual functions of TR for transcriptional regulation of T3 inducible genes exist *in vivo* during *Xenopus* development, involving changes in histone modification and chromatin remodeling, including the removal of nucleosomes at TREs to likely generate open chromatin for transcriptional activation when T3 is present.

The biological significance of the dual functions of TR was first demonstrated with transgenic approach initially established for *Xenopus laevis.* Transgenic expression of a dominant negative TR that cannot bind T3 and functions as a constitutive repressor of T3-inducible genes inhibited metamorphosis (23, 32–35). In contrast, inducible expression of a transgenic dominant positive TR that cannot bind T3 but functions as a constitutive activator, resembling liganded TR, of T3-inducible genes induced precocious metamorphosis in premetamorphic tadpoles (36–38). This is accompanied by similar regulation of T3 response genes, demonstrating a critical role of TR for metamorphosis. Furthermore, transgenic expression of a dominant negative corepressor N-CoR, which binds to unliganded TR to facilitate repression of T3-inducible genes, led to depression of TR-target genes in premetamorphic tadpoles and premature initiation of metamorphosis (39), again supporting the model. Complementary to this, transgenic expression of a dominant negative form of SRC3, a coactivator that binds to liganded TR, inhibited both T3-induced activation of TR-target genes in premetamorphic tadpoles and T3-induced or natural metamorphosis (26). Similar observations were made with transgenic expression of another coactivator, p300, which binds to SRC3 to form a large complex, revealing the importance of intact coactivator complexes containing SRC3 or related proteins (SRC1 and SRC2) in gene activation by liganded TR and *Xenopus* metamorphosis (27). Finally, transgenic expression of another coactivator, PRMT1, a histone methyltransferase that can form a coactivator complex with SRC3-p300, enhanced gene activation by liganded TR and accelerated metamorphosis (24). Thus, unliganded TR recruits corepressors to regulate metamorphic timing while liganded TR recruits coactivators to control the rate of metamorphosis progression during *Xenopus* development, as predicted by the dual function model for TR.

## Gene knockout studies of the function of endogenous TRs

The development of gene-editing technologies made it possible to knockout genes in both *Xenopus laevis* and *Xenopus tropicalis*, with the latter more advantageous for genetic and genome-wide studies due to its diploid genome. The first knockout (KO) studies were carried out on TRα (40–47), whose expression profile suggests its role both during premetamorphosis and during metamorphosis. Indeed, knocking out TRα led to precocious initiation of metamorphosis (i.e., reaching the onset of metamorphosis (stage 54) at younger age), but delayed progression of metamorphosis (taking longer time to reach the climax of metamorphosis from stage 54) without significantly affecting the overall time from fertilization to the end of metamorphosis (stage 66). These findings demonstrate that endogenous TRα indeed has dual functions during frog development as the model predicted.

**Figure 2:**
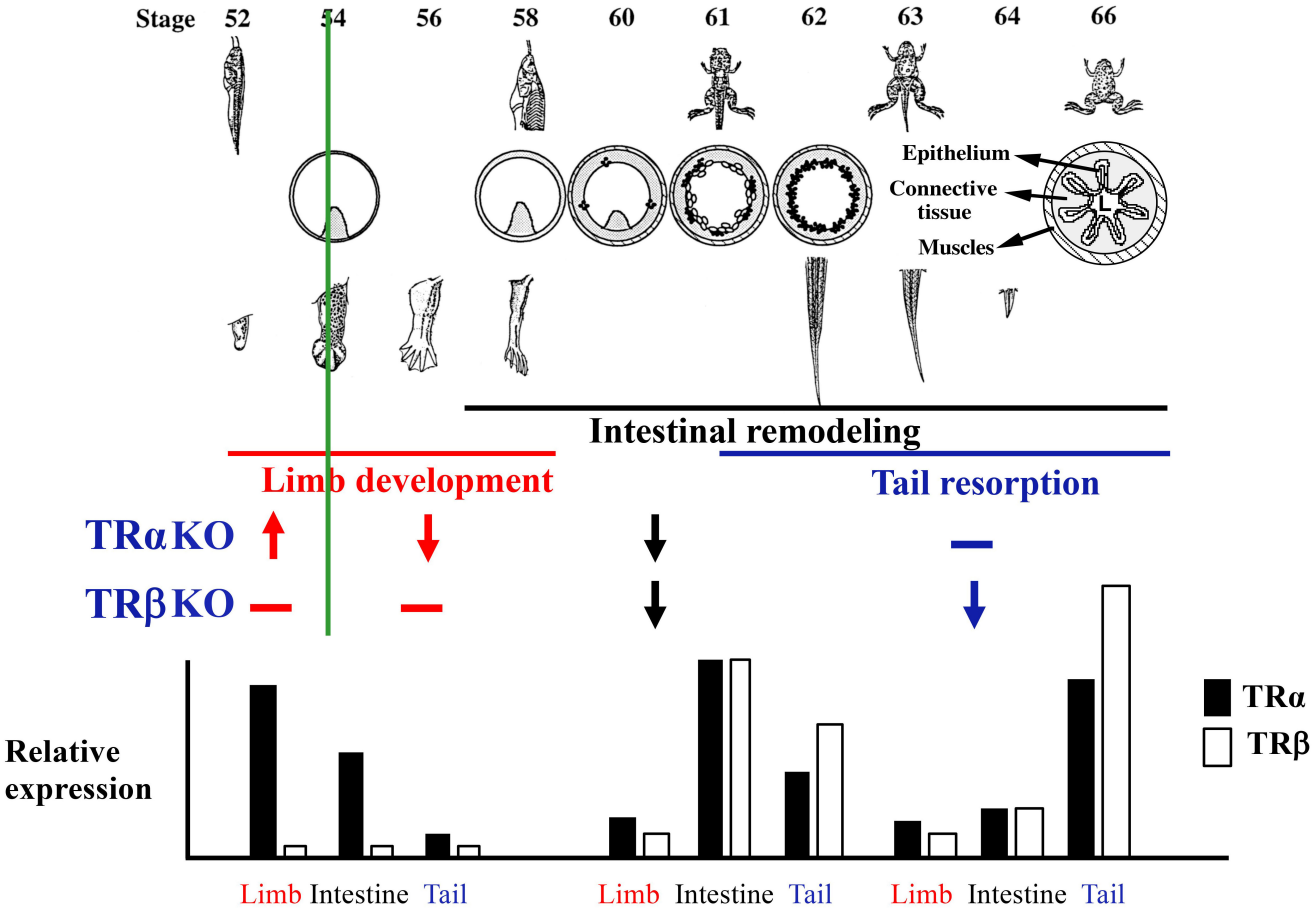
Distinct organ-specific effects of TRα- and TRβ-KO correlate with relative TR expression in the organs during development. Upper: Morphological changes of the whole animal, intestine, limb, and tail during metamorphosis. Middle: Knocking out TRα or TRβ has stage-dependent effects on metamorphosis of the limb, intestine, and tail. TRα KO accelerates (arrow pointing up) limb development prior to the onset of metamorphosis (stage 54, green line) but inhibits (arrow pointing down) it after the onset of metamorphosis, inhibits intestinal remodeling between stage 58 and stage 66, but has no effect (-) on tail resorption between stage 61 and stage 66. In contrast, TRβ KO does not affect limb development but inhibits intestinal remodeling and tail resorption (see (49) for more details). Note that these phenotypes are based on KO studies in *Xenopus tropicalis*, although similar results are expected for *Xenopus laevis.* Lower: relative expression of TRα and TRβ in the limb, intestine, and tail around stage 54 (left, premetamorphosis), stage 61 (a climax stage when intestinal remodeling is occurring dramatically), and stage 63 (a climax stage when tail resorption is occurring rapidly). Based on (56, 57).

Interestingly, analyses of animals lacking TRα or TRβ revealed distinct phenotypes caused by TRα or TRβ KO (40–44, 48, 49). There are three major types of changes during metamorphosis, exemplified by the hindlimb, intestine and tail (**Figure 2**). The hindlimb undergoes *de novo* development, beginning prior to the onset of metamorphosis at stage 54 (a stage defined based on mainly the ability to detect plasma T3) with morphogenesis mostly occurring between stage 54 and stage 58 (when hindlimb development is largely complete). Intestinal remodeling occurs mostly during metamorphic climax (stage 58 to stage 66) when larval epithelium undergoes apoptotic degeneration and adult epithelium develops through formation of adult progenitor/stem cells followed by their proliferation and differentiation. Finally, tail resorption occurs mainly after stage 61 when tail length reduction occurs rapidly through apoptosis with the complete resorption of tail marking the completion of metamorphosis at stage 66. For the limb, TRα KO accelerated its development before stage 54 but delayed it between stage 54 to stage 58, while TRβ KO had no significant effect on limb development at morphological level (**Figure 2**). On the other hand, both TRα KO and TRβ KO delayed intestinal remodeling. In contrast to the limb, tail resorption between stage 61 and stage 66 was not affected TRα KO, but was inhibited by TRβ KO inhibited, particularly the resorption of the notochord. Thus, the endogenous TRα and TRβ have distinct, organ- and developmental stage-dependent functions during *Xenopus* development.

Finally, knocking out of both TRα and TRβ leads to tadpole death at the climax of metamorphosis (stage 61) (50, 51). These TR double KO tadpoles have most or all adult organs formed, often, precociously, but fail to resorb the larval specific tissues/organs including the larval intestinal epithelium, gills, and tail. Thus, TR is essential for the completion of metamorphosis and tadpole survival.

## Temporal regulation of TRα and TRβ expression appears to dictate the roles of endogenous TR through target binding in different organs during development

Insight into the mechanisms underlying the distinct effects of TRα and TRβ KO first came from earlier data on the expression of the two genes (**Figure 2**) (52–56). Around the onset of metamorphosis, TRα is expressed at a much higher level in the limb than in the tail with the level in the intestine in-between the two organs, while TRβ expression is very low in all three organ (**Figure 2**). The lack of TRβ expression at this early stage may explain why TRβ KO does not affect limb development. The high level of TRα expression underlie the observations that TRα KO accelerates limb development prior to metamorphosis, likely due to de-repression of TR target genes caused by unliganded TRα, but inhibits limb development after stage 54 due to reduced signaling by T3 upon TRα KO. Both TRα and TRβ have peak levels of expression around stage 61 in the intestine when most dramatic intestinal remodeling, including larval cell death and adult cell proliferation, occurs. This may underlie the fact that KO of either TRα or TRβ inhibits intestinal remodeling. In the tail, the expression of TRβ is drastically upregulated to even higher levels than that of TRα by metamorphic climax (52, 54–56). This may explain why TRβ KO inhibits tail resorption. It is possible that the TRβ level is sufficiently high in the tail by metamorphic climax that tail resorption can occur without TRα. Finally, it is also interesting to note that TR expression is the highest in an organ at the stages when metamorphosis of the organ occurs, i.e., around stage 54 in hindlimb, stage 61 in the intestine, and stage 63 in the tail (52, 55, 56), further supporting an important regulation of TR function through controlling TR expression.

**Figure 3:**
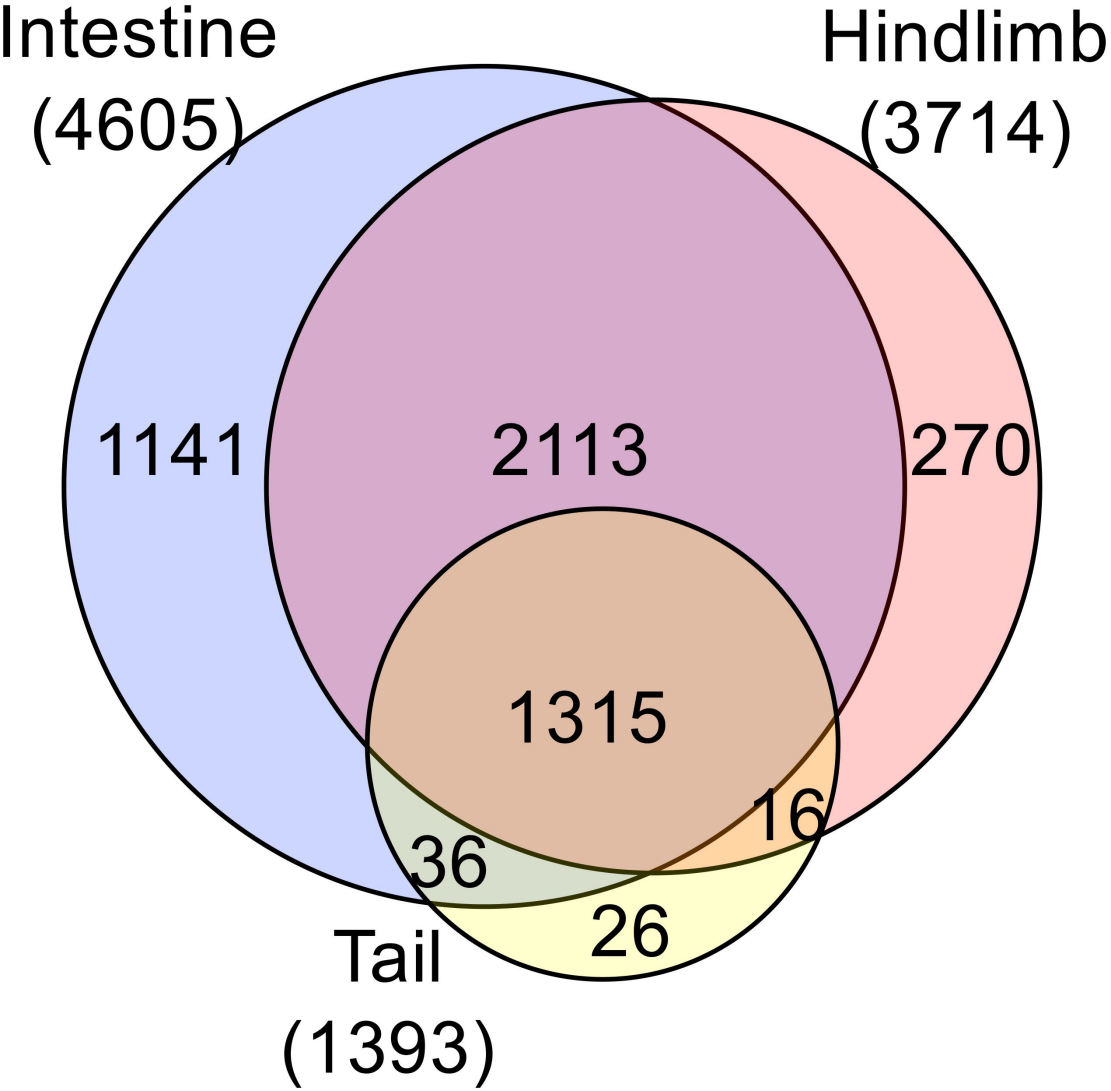
Comparative analysis of ChIP-seq data reveals much fewer TR-bound genes in the tail compared to the limb and intestine at the onset of metamorphosis. Venn diagram analysis was performed for TR-bound genes as obtained from ChIP-seq data for the tail, intestine and hindlimb of premetamorphic tadpoles treated with or without T3. Note that most TR-bound genes are common among the organs. The tail has the smallest proportion of unique TR-bound genes (26 out of 1393 or 2%). The ChIP-seq were performed on organs pooled from multiple premetamorphic tadpoles at stage 54 with or without T3 treatment. See (57) for more details.

The development of chromatin-immunoprecipitation sequencing (ChIP-seq) made it possible to analyze genome-wide binding of TR to target genes in different organs. ChIP-seq analyses with an antibody recognizing both TRα and TRβ revealed that in premetamorphic tadpoles, the number of genes bound by TR in the hindlimb, intestine, and tail were higher when tadpoles were treated with T3 for 1 day compared to control tadpoles (57–59), likely due to 1) increased TR expression, particularly TRβ, which is a TR target gene, and/or 2) increased binding of TR to target in the presence of T3. Importantly, the number of genes bound by TR in the presence and/or absence of T3 were much higher in the intestine and hindlimb than that in the tail (**Figure 3**), consistent with the much lower levels of TR expression in the tail compared to those in the hindlimb and intestine around stage 54 (**Figure 2**). Interestingly, among the TR-bound genes, most were common among the three organs despite the very different metamorphic changes in these organs during metamorphosis. This suggests that the small fractions of organ-specific TR target genes specify organ-specific metamorphosis and/or common TR target genes function in conjunction with genes which are not direct TR target genes but expressed in an organ-specific manner to control specific changes in different organs during metamorphosis.

## Endogenous TRα and TRβ bind mostly the same target genes and their binding correlates with their expression levels

**Figure 4:**
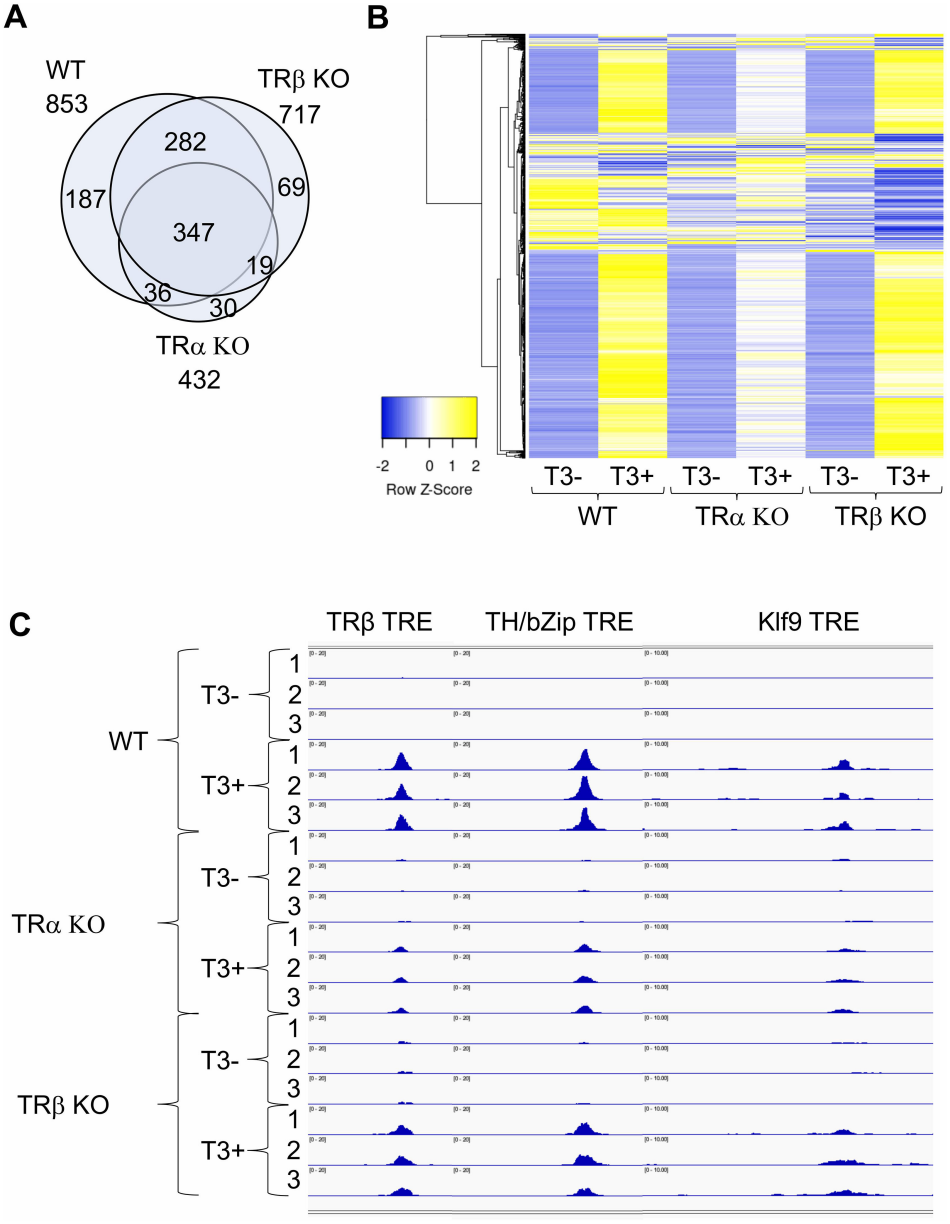
There are few TRα- or TRβ-specific TR-bound genes in intestine and TRα KO has bigger effect on target binding than TRβ KO in the intestine of premetamorphic tadpoles. **(A)** Venn diagram showing overlap among TR-bound genes identified in the wild type (WT), TRα KO, and TRβ KO intestine. The TR-bound genes in the presence or absence of T3 treatment for each genotype were combined together for the comparison. Note that 1) most genes bound by TR were common among all three genotypes, suggesting that TRα and TRβ bind to the same genes. There were more genes bound by TR in TRβ KO intestine compared to TRα KO intestine, suggesting that TRα KO has a bigger effect on TR-binding to targets than TRβ KO, presumably due to the higher level of TRα expression at stage 54. **(B)** Heatmap showing the CPM (count per million) value for all TR-bound genes identified in WT, TRα KO, and TRβ KO intestine with or without T3 treatment. CPM values were normalized across rows and clustered with Euclidean distance metric and average linkage. Blue to yellow gradient represents normalized level of TR binding (CPM) from low to high. Note that there are more genes with increased TR-binding (blue to yellow) after T3 treatment of WT and TRβ KO tadpoles compared to TRα KO tadpoles, indicating that TRα KO has a bigger effect on TR-binding to individual targets than TRβ KO. **(C)** TR binding to known TREs in the intestine of WT, TRα KO, and TRβ KO tadpoles at stage 54 increased after T3 treatment. IGV software was used to visualize known TRE regions of TR-target genes TRβ, TH/bZip, and Klf9, in the ChIP-seq data. Blue peaks in each wiggle plot represent the normalized ChIP-seq reads. There were three technical replicates for each sample (-T3 or +T3). The ChIP-seq were performed on intestines pooled from multiple premetamorphic tadpoles at stage 54 with or without T3 treatment. See (60) for more details.

TRα and TRβ are highly conserved with the major sequence differences located at the N-terminus before the DNA binding domain. TR’s ability to form heterodimer with RXR, bind to ligand, and regulate transcription depends on the sequence from DNA-binding domain to the C-terminus, although the N-terminus can contribute to TR function. Thus, it is possible that the distinct effects of TRα KO and TRβ KO may be due to the sequence difference at the N-terminus to affect target DNA binding. The availability of TRα KO and TRβ KO tadpoles made it possible to investigate at the genome-wide level the effects of individual TR KO on target binding. By using a quantitative ChIP-seq analyses, which expresses TR binding to individual sites as counts per million ChIP-seq reads with a minimal count cutoff set for TR-bound site, we analyzed TR binding in the intestine of wild type, TRα KO, and TRβ KO premetamorphic tadpoles treated with or without T3 (60). While the analyses yielded fewer TR-bound genes compared to traditional ChIP-seq (compare WT data in **Figure 4A** to the intestine data in **Figure 3**, presumably duo to more stringent minimal count cutoff in the quantitative ChIP-seq), it offered a quantitative comparison of the effects of TRα KO and TRβ KO. Consistent with the high levels of TRα expression while very low levels of TRβ expression in premetamorphic tadpole intestine (**Figure 2**, stage 54), TRα KO drastically reduced the number of TR-bound genes while TRβ KO had very little effect (**Figure 4A**). When a heatmap of TR-binding in the presence and absence of T3 was generated on all TR-bound genes in the intestine of all three genotypes, it showed that at the level of individual TR target sites (**Figure 4B**), T3 enhanced or reduced TR-binding to targets dramatically for the vast majority of the genes. TRα KO significantly reduced the effects of T3 while the heatmap for TRβ KO intestine was largely similar to that of the wild type intestine (**Figure 4B**). Interestingly, a small fraction of genes whose binding by TR seemed to be more reduced by T3 treatment in the TRβ KO compared to WT. It is unclear why this was the case, but it could suggest that TRβ binding to these genes may be more difficult to be disrupted by T3 compared to TRα binding, and thus in the absence of TRβ, T3 would cause a bigger reduction in TR binding since only TRα is bound to these genes.

The differential effects of TRα and TRβ KO on target binding are more clearly illustrated by TR binding to the three well known TR targets, TRβ, TH/bZip, and Klf9, in the presence or absence of T3 treatment of wild type, TRα KO, and TRβ KO tadpoles, where the binding peaks for all genes were enhanced by T3 treatment in wild type intestine and this enhancement was slightly reduced by TRβ KO but drastically reduced by TRα KO (**Figure 4C**). Thus, TRα and TRβ bind to mainly the same TR-target genes and the extent of binding to individual target genes is mainly determined by TR expression.

## Discussion

For over a century, anuran metamorphosis has served as a great model for studying postembryonic vertebrate development, particularly the roles of T3 signaling and the underlying molecular mechanisms. The cloning of TR and demonstration of TR being DNA-binding transcription factors opened up molecular and genetic studies of the roles of TR during development in *Xenopus laevis* and *Xenopus tropicalis*, which are highly related anuran species but offer complementary advantages. These studies have demonstrated that TRs have dual functions during anuran development, controlling developmental timing in the unliganded form, mainly by TRα, which is expressed at high levels prior to the onset of metamorphosis and regulating metamorphic rate when liganded by T3. Recent gene KO studies indicate the endogenous TRα and TRβ have distinct, developmental stage-dependent roles during metamorphosis. Interestingly, while individual TR gene is not required for *Xenopus* development and reproduction, both TRα and TRβ affects the rate of development in organ- and stage-dependent manners and TR is essential to ensure tadpole survival at the climax of metamorphosis and thus the completion of metamorphosis.

Molecularly, consistent with the high conservations between TRα and TRβ, both TRs appear to bind to mostly the same target genes, at least in the tadpole intestine. Furthermore, the effects of individual TR KO correlate well with the relative expression of TRα and TRβ in different organs during development. Finally, organs with high levels of TR expression have a larger number of TR-bound genes, at least for premetamorphic tadpoles. Thus, distinct roles of endogenous TRα and TRβ appears to be dictated largely by the spatiotemporal expression of the TRα and TRβ genes. On the other hand, it is still possible that the sequence differences between TRα and TRβ, particularly at the N-terminus, may lead to certain less obvious but distinct roles during development that are yet to be discovered.

Many of the findings on TR during *Xenopus* development appear to be conserved with findings on TR during mouse development. During postembryonic (peri- and post-natal) development in mammals when plasma T3 level peaks, many organs undergo maturation into the adult form, resembling adult organ development during *Xenopus* metamorphosis (1, 4, 61). For example, the mouse intestine matures into the complex adult form with *de novo* formation of adult stem cells and the crypt, where the stem cells reside. There is also a transition from embryonic to adult hemoglobin to adapt to air-breathing after birth when the lung matures. The heart, likewise, undergoes maturation with the heart rate changes after birth when T3 level rises. Studies on TR expression and function in mouse also support critical roles of TR in these processes. Like in *Xenopus*, TRα is also expressed early during mouse embryogenesis while TRβ is activated much later (62, 63). The expression of TRs, particularly TRα, during early development when plasma T3 level is low, in mouse, just like in *Xenopus*, also suggest a role of unliganded TR during mouse development. Consistently, TRα knockout increases the expression of several T3-response genes in the heart, as well as increased heart rate of the embryos (64). After birth, when T3 levels rises, these T3-response genes are expressed at lower level in TRα knockout mice compared to the wild type mice and the heart rate is also reduced in TRα knockout mice, opposite of those observed in the embryos. These supports a dual function model for TRα, i.e., repressing the target genes and keeping the heart rate low in the unliganded state in the embryos and does the opposite as liganded TR after birth when T3 level is high. Furthermore, in both mouse and *Xenopus*, most adult organs/tissues can form in animals with both TRα and TRβ knocked out (62, 63, 65–69). In both species, there are many developmental abnormalities in the absence of TR. Furthermore, the effects of knocking out individual TR subtypes on different mouse organs also correlate with the relative expression levels of TR subtypes. The most noticeable difference between TR KO mice and *Xenopus* is that *Xenopus* tadpoles without TR cannot complete metamorphosis and die at the climax of metamorphosis while mouse without any TR can develop into adult, albeit with abnormalities. In this regard, it is worth noting that zebrafish can also develop into adults without any TR, although also with defects in various organs (70). This essential role of TR for tadpole metamorphosis and survival may be related to the fact that a major fraction of the tadpole, including the gills and tail, needs to be resorbed to complete metamorphosis. This resorption process requires TR. Thus, the reason for the different survival outcomes of animals without TR in *Xenopus*, zebrafish, and mouse may be due to the need for coordinated removal of larval organs, which is mostly absent in mouse and zebrafish but essential for *Xenopus*.

Currently, little is known on the role of endogenous TRs during development in species other than *Xenopus tropicalis* and mouse. It would be valuable to analyze the phenotypic and molecular effects of TR KO effects in other species, such as other amphibians and mammals, and older vertebrate classes like teleost. Of particular interest is the flatfish, whose metamorphosis includes perhaps the most dramatic structural remodeling among fishes, the migration of one eye to the opposite side of the head (71). Flatfish metamorphosis also involves larval cell death and is controlled by T3 (71–74). Fish also has both TRα and TRβ and TRα is expressed at high levels during premetamorphosis when little T3 is present (71, 75–78). Thus, it is very likely that TRs have dual functions during flatfish development. Molecular and genetic studies, particularly TR knockout, in flatfish should definitively reveal the roles of TRs in fish development. Such studies in different animal species may not only determine any conservation in TR function during evolution but also provide information on whether TR may play an active role to facilitate vertebrate evolution.
